# Emerging Pathogen in Wild Amphibians and Frogs (*Rana catesbeiana*) Farmed for International Trade

**DOI:** 10.3201/eid0908.030030

**Published:** 2003-08

**Authors:** Rolando Mazzoni, Andrew A. Cunningham, Peter Daszak, Ada Apolo, Eugenio Perdomo, Gustavo Speranza

**Affiliations:** * Instituto de Investigaciones Pesqueras, Montevideo, Uruguay; † Institute of Zoology, London, UK; ‡ Consortium for Conservation Medicine, Palisades, New York, USA

**Keywords:** frogs, wild amphibians, chytridiomycosis, dispatch

## Abstract

Chytridiomycosis is an emerging disease responsible for global decline and extinction of amphibians. We report the causative agent, *Batrachochytrium dendrobatidis*, in North American bullfrogs (*Rana catesbeiana*) farmed for the international restaurant trade. Our findings suggest that international trade may play a key role in the global dissemination of this and other emerging infectious diseases in wildlife.

Cutaneous chytridiomycosis is an emerging fungal disease of amphibians responsible for a mass die-off, population decline, and extinction of amphibians on a global scale (*1*,*2*). In wild, susceptible species, chytridiomycosis may be able to cause catastrophic population loss, sometimes completely removing local populations (*2*). This disease is a serious threat to the conservation of wild amphibians, and policy measures to control amphibian movements have been established by at least one authority (Parks and Wildlife Commission of the Northern Territory, Australia; Bill Freeland, pers. comm.). Chytridiomycosis is caused by a zoosporic fungus, *Batrachochytrium dendrobatidis*, which develops solely within keratinized cells (*3*), causing extensive hyperkeratosis and death by an, as yet, unknown mechanism (*1*,*4*,*5*).

Chytridiomycosis is a key example of an emerging infectious disease in wildlife (*6*,*7*). The most important factor driving the emergence of such wildlife diseases is the anthropogenic introduction of pathogens into new geographic areas (pathogen pollution) (*6*–*9*). We report on *B. dendrobatidis* in captive bullfrogs, which was identified during an episode of unusually high death rates of unknown cause and which implicates a relatively new food animal trade in the spread of this disease.

## The Study

Mass deaths occurred at a large rearing facility for North American bullfrogs (*Rana catesbeiana*), 46 km from Montevideo, Uruguay. The farm is a commercially managed operation, with trained employees under permanent, technical supervision. The farm produces approximately 150,000 tadpoles each summer and 30,000 metamorphs. Mortality rates during the days before the outbreak were approximately 0.5% per week, which is considered normal for this type of farm. Frog growth rates were also within normal parameters for these conditions (*10*).

At this farm, spawning occurs in 50-L tanks from October to February (spring and summer), tadpoles grow until March, and metamorphosis occurs from March to May (fall season). Newly metamorphosed frogs (5–8 g) are harvested from tadpole ponds and stocked in tanks with circulating water until resorption of the tail is complete. Frogs are then transferred to large growing tanks, in which the frogs are reared at a density of 500 per square meter while standing in shallow water with only their bodies immersed (*10*). Permanently running water is piped into the system from a well on the farm. Water temperature ranges from 17°C to 19°C, and pH is stable at 7.1. Frogs are fed with trout chow (Purina Aquamax Carnivorous) and earthworms (*Eisenia foetida*) cultured at the farm. Tanks are cleaned every day to eliminate excess feed and feces, and animals are sorted to maintain homogeneous sizes in each tank. Dead animals are removed twice a day.

The mass die-off began on May 1, 1999. Earlier in the same week, a tank containing 5,000 recently metamorphosed frogs had been emptied, and the population placed in three other tanks along with other frogs that had metamorphosed during the same week. The following day, frogs started to die in unusually high numbers (300 deaths on day 1) in these three tanks. To reduce the risk of bacterial infections, poly-hexamethylene-biguanide-hydrochloride (Vantocil IB, Zeneca Biocides, Blackley, Manchester, UK) was added to the tanks to give a final concentration of 500 ppm. The deaths continued and spread to other tanks; 15,000 frogs died during the first week. An antifungal therapy (benzalkonium chloride 2 ppm baths, “Farmazul” 1% solution, Lab. Farmaco-Uruguayo, Acuña de Figueroa, Montevideo) (*11*) was used, but this treatment was also ineffective and almost 95% of the farm’s total population of recently metamorphosed frogs died within 30 days. Mortality rates in individual tanks were extremely high, with affected tanks losing 10% of the population on day 1 and usually >90% of the population by day 3 or 4. By day 10, all frogs in the three index tanks were dead, and the disease had spread to metamorphs in other tanks on the farm. The epidemic continued for 26 days (until May 27), when only 2,000 of the original 30,000 metamorphs remained. None of the broodstock was affected, even though these animals were in close spatial association with the affected tanks.

Affected frogs became lethargic with visible accumulations of sloughed skin. Although nonspecific, these signs, the high mortality rate, and the age of affected frogs are consistent with those reported for chytridiomycosis (*2*). Many of the frogs had agonal convulsions and extension of hind limbs; death occurred 12–24 h later. Reddening of ventral skin was found in some cases a few hours before death, usually in the pelvic and femoral areas. Tadpoles showed no signs of disease or increased death rates.

Routine bacteriologic tests (culture on blood agar for 24 h and 48 h at 37°C) were performed on liver, kidney, and heart blood collected from a sample of euthanized frogs, but these tests did not implicate causative pathogens. Necropsies were performed systematically on 30 frogs, but no gross abnormalities of the viscera were found. Internal organs (lung, liver, kidney, heart, and spleen) from 10 of these frogs were examined by histologic testing, but no lesions were detected. Examination of skin smears (wet-mounted, stained with a 1:1 mixture of cotton blue [parker ink] and 10% aq. KOH), and histologic and ultrastructural analyses of fixed skin indicated subspherical developing sporangia and flask-shaped mature sporangia within the superficial layer of the epidermis (Figure). Septa, characteristic of *B. dendrobatidis*, were observed in many developing sporangia (Figure). Slight hyperplasia of the keratinized cells of the stratum corneum and stratum granulosum was observed in some areas where chytrid developmental stages were present. Specific examinations for viruses were not performed, as facilities to test for viruses were unavailable.

**Figure:**
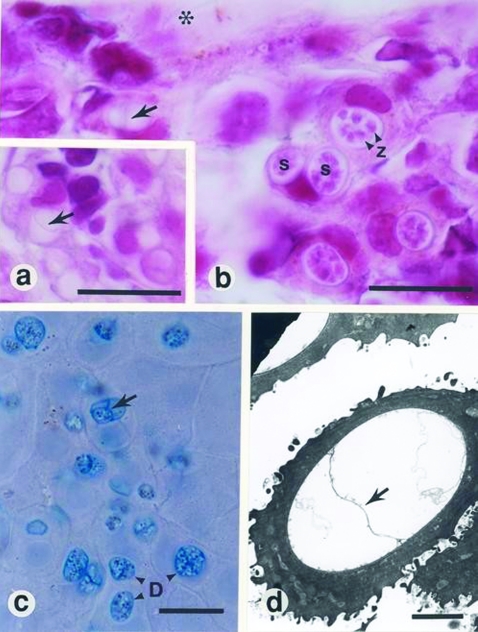
a and b, histopathologic findings from infected frogs. Characteristic sporangia (s) containing zoospores (z) are visible in the epidermis (asterisk, superficial epidermis; arrow, septum within an empty sporangium; bars, 10 μm. c, Skin smear from infected frog, stained with 1:1 cotton blue and 10% aqueous potassium hydroxide (aq KOH) (D, developing stages of *Batrachochytrium dendrobatidis*; arrow, septum within a sporangium; bar, 10 μm. d, Electron micrograph of an empty sporangium showing diagnostic septum (arrow) (bar, 2 μm).

In a basic transmission experiment, two groups of five apparently healthy frogs from affected tanks were placed in separate aquaria, one contained five frogs from an unaffected farm in Uruguay and the other empty. Deaths began on day 4, with death occurring 24–48 h after first signs of disease. Within 8 days, all 15 frogs were dead. A group of five control frogs from the unaffected farm remained healthy.

After the incident, death rates returned to levels before the event. As a result of this event, monthly surveillance for cutaneous chytrid infection was instigated; ventral hind limbs, feet, and digits are examined from five healthy frogs and from any sick or dead frogs. *B. dendrobatidis* has been repeatedly observed in skin smears of healthy frogs from the farm at which the outbreak occurred. Similar results have since been obtained from six other bullfrog farms in Uruguay (R. Mazzoni, unpub. data). Histologic examination of infected frogs on which necropsies were performed since the 1999 epidemic shows focal mild hyperplasia of the superficial epidermis associated with sporangia of *B. dendrobatidis*. No unusual death rates have occurred at the index site since the 1999 incident.

## Conclusions

Cutaneous chytrid infection was detected during an investigation into mass deaths in farmed North American bullfrogs. Although chytridiomycosis has been reported as an important cause of mass deaths in wild amphibians, whether chytrid infection was the cause of deaths in the disease outbreak described in this paper is unclear. First, while no examinations for *B. dendrobatidis* had been performed before the deaths, this organism has been repeatedly observed in skin examinations of healthy frogs in follow-up studies. Second, although not proved efficacious against cutaneous chytridiomycosis, the use of benzalkonium chloride, a recognized therapy for infections with nonhyphal fungi, failed to reduce mortality rates.

Possible causative agents include an infectious pathogen, which could not be detected by the examinations used, such as a ranavirus (*2*) or adverse environmental factors. Alternatively, chytridiomycosis could be the cause of the outbreak if the current chytrid is a different strain from that causing the outbreak or became attenuated, if the farmed bullfrogs had immunity to the pathogen, or if a change in the environment before the die-off allowed a normally benign infection to become pathogenic. For example, increased temperatures reversibly inhibit both growth of *B. dendrobatidis* in culture (*3*) and the progress of disease outbreaks (D. Nichols, Smithsonian Institute, pers. commun.). Also, chytridiomycosis epizootics in wild U.S. amphibians often coincide with late-winter breeding (*2*). The deaths we describe occurred at the beginning of winter and may have been precipitated by lowered environmental temperatures.

The mortality rates reported here were dramatic and costly. The rearing of bullfrogs is a growth industry in South America, particularly Brazil, Uruguay, and Argentina. Anecdotal reports of similar loss of stock in a number of farms in Uruguay (R. Mazzoni, unpub. data) suggest that disease is a major economic threat to this industry in South America. This situation is exacerbated by a lack of veterinary diagnostic capacity, management protocols, and treatment or prophylaxis.

Regardless of the cause of the deaths, the North American bullfrog may be relatively resistant to chytridiomycosis, at least under the environmental conditions in the current study. Unpublished data suggest that metamorph bullfrogs can be experimentally infected by *B. dendrobatidis* without developing signs of disease (*12*), and others have noted differential susceptibility to this disease among amphibian species (*1*,*2*).

Nonclinical (silent) infections in farmed bullfrogs suggest that this species may act as a carrier of chytridiomycosis, which has serious implications for conservation of biodiversity. *B. dendrobatidis* has been implicated in the complete removal of multiple species amphibian populations over large geographic areas in the wild (*1*,*2*). Farmed bullfrogs originate in North America and have been introduced to South America during the last few decades for the lucrative restaurant trade. Live, farmed South American frogs are exported to other South American countries and to the United States. The trade in bullfrogs is large in scale and global in scope. According to the U.S. Fish and Wildlife Service, over 1 million bullfrogs are imported into the United States each year from South America alone (*9*), with others shipped in from Asia. In the United States, animals are usually imported live at U.S. ports of entry and undergo veterinary inspections that are inadequate for identifying chytrid infection.

Recently, guidelines for amphibian translocations have been published that include specific quarantine and testing recommendations for chytridiomycosis (*13*). These procedures, however, are intended for animal movements for International Union for Conservation of Nature and Natural Resources programs and are not legally binding. In the United States, the U.S. Department of Agriculture (USDA) may be the most suitable regulatory body for the bullfrog trade since these animals are a commercial agricultural product. No current USDA regulations exist for the identification of infectious agents in amphibians.

The farm we studied was opened in October 1998 with stock that originated from Brazil. The Brazilian farmers originally imported 300 pairs of frogs from Canada to set up the first South American farms approximately 30 years ago. The pathogen has been present in bullfrogs in North America since at least the 1970s (*14*). *B. dendrobatidis* in this outbreak may have been imported with breeding stock from North America. Alternatively, wild South American amphibians may be the source of infection. The Uruguay farm in our study is enclosed by concrete walls, but outdoor ponds have open access to wildlife, and wild amphibians (tree frogs) have been observed in the greenhouses where indoor rearing tanks are located. Whatever the origins of *B. dendrobatidis* in farmed bullfrogs in Uruguay, such open-plan farms are likely sources of infection and disease for wild amphibian species. In view of this risk, surveillance for chytrid infection and for unusual levels of deaths in endemic amphibian species should be established in the vicinity of frog farms in Uruguay and elsewhere in the world.

Global trade and commerce are regularly cited as drivers of disease emergence in humans, domestic animals, wildlife, and plants (*6*,*15*–*18*). For wildlife emerging infectious diseases, quantitative analyses demonstrate that this process is the most important driving factor (*8*). For example, the (unknown) causative agent of the bullfrog deaths we describe may be spread by such trade. Also, the identification of *B. dendrobatidis* in an international food animal trade has implications for amphibian conservation and for disease emergence in general. Our study adds to reports of chytridiomycosis in the international pet trade (*19*), amphibians for outdoor pond stocking in the United States (*2*,*20*), importation for zoo collections (*5*), trade in laboratory animals (*21*), and species known to have recently been introduced into new geographic regions (e.g., cane toads in Australia) (*1*).

With a continued rise in the international air transport volume (*22*), we predict a growing impact of trade and commerce on disease emergence within all populations, including wildlife. For this reason, we urge a revision of national and international veterinary guidelines for the inspection and quarantine of imported animals and animal products.

## References

[1] Berger L, Speare R, Daszak P, Green DE, Cunningham AA, Goggin CL, Chytridiomycosis causes amphibian mortality associated with population declines in the rainforests of Australia and Central America. Proc Natl Acad Sci U S A. 1998;95:9031–6. 10.1073/pnas.95.15.9031 9671799 PMC21197

[2] Daszak P, Berger L, Cunningham AA, Hyatt AD, Green DE, Speare R. Emerging infectious diseases and amphibian population declines. Emerg Infect Dis. 1999;5:735–48. 10.3201/eid0506.990601 10603206 PMC2640803

[3] Longcore JE, Pessier AP, Nichols DK. *Batrachochytrium dendrobatidis* gen. et sp. nov., a chytrid pathogenic to amphibians. Mycologia. 1999;91:219–27. 10.2307/3761366

[4] Nichols DK, Lamirande EW, Pessier AP, Longcore JE. Experimental transmission of cutaneous chytridiomycosis in dendrobatid frogs. J Wildl Dis. 2001;37:1–11. 11272482 10.7589/0090-3558-37.1.1

[5] Pessier AP, Nichols DK, Longcore JE, Fuller MS. Cutaneous chytridiomycosis in poison dart frogs (*Dendrobates* spp.) and White’s tree frogs (*Litoria caerulea*). J Vet Diagn Invest. 1999;11:194–9. 10098698 10.1177/104063879901100219

[6] Daszak P, Cunningham AA, Hyatt AD. Emerging infectious diseases of wildlife—threats to biodiversity and human health. Science. 2000;287:443–9. 10.1126/science.287.5452.443 10642539

[7] Daszak P, Cunningham AA, Hyatt AD. Anthropogenic environmental change and the emergence of infectious diseases in wildlife. Acta Trop. 2001;78:103–16. 10.1016/S0001-706X(00)00179-0 11230820

[8] Dobson A, Foufopoulos J. Emerging infectious pathogens of wildlife. Philos Trans R Soc Lond B Biol Sci. 2001;356:1001–12. 10.1098/rstb.2001.0900 11516378 PMC1088495

[9] Cunningham AA, Daszak P, Rodríguez JP. Pathogen pollution: defining a parasitological threat to biodiversity conservation. J Parasitol. 2003. In press.

[10] Mazzoni R, Carnevia D, Altieri W, Matsumura Y. Cría de ranas en Sistema Inundado Experiencias en ranarios comerciales. Bol Inst Invest Bibliogr (Mexico). 1996;13:43–51.

[11] Groff JM, Mughannam A, McDowell TS, Wong A, Dykstra MJ, Frye FL, An epizootic of cutaneous zygomycosis in cultured dwarf African clawed frogs (*Hymenochirus curtipes*) due to *Basidiobolus ranarum.* J Med Vet Mycol. 1991;29:215–23. 10.1080/02681219180000331 1941429

[12] Daszak P, Cunningham AA, Hyatt AD. Infectious disease and amphibian population declines. Divers Distrib. 2003;9:141–50. 10.1046/j.1472-4642.2003.00016.x

[13] Cunningham AA, Daszak P, Hyatt AD. Amphibia. In: Quarantine and health screening protocols for wildlife prior to translocation and release into the wild. Woodford MH, editor. Paris: Office International des Epizooties; 2001. p. 74–9.

[14] Green DE, Kagarise-Sherman C. Diagnostic histological findings in Yosemite toads (*Bufo canorus*) from a die-off in the 1970s. J Herpetol. 2001;35:92–103. 10.2307/1566028

[15] Lederberg J, Shope RE, Oakes SC. Emerging infections: microbial threats to health in the United States Institute of Medicine. Washington: National Academy Press;1992. 25121245

[16] Morse SS. Examining the origins of emerging viruses. In: Morse SS, editor. Emerging viruses. New York: Oxford University Press; 1993. p. 10–28.

[17] Islam MA, Rahman MM, Adam KH, Marquardt O. Epidemiological implications of the molecular characterization of foot-and-mouth disease virus isolated between 1996 and 2000 in Bangladesh. Virus Genes. 2001;23:203–10. 10.1023/A:1011821027235 11724275

[18] Anderson PK, Morales FJ. The emergence of new plant diseases: the case of insect-transmitted plant viruses. Ann N Y Acad Sci. 1994;740:181–94. 10.1111/j.1749-6632.1994.tb19868.x 7840449

[19] Mutschmann F, Berger L, Zwart P, Gaedicke C. Chytridiomykose bei amphibien-erstmaliger nachweiss fur Europa. Berl Munch Tierarztl Wochenschr. 2000;113:380–3. 11084755

[20] Parker JM, Mikaelian I, Hahn N, Diggs HE. Clinical diagnosis and treatment of epidermal chytridiomycosis in African clawed frogs (*Xenopus tropicalis*). Comp Med. 2002;52:265–8. 12102573

[21] Reed KD, Ruth GR, Meyer JA, Shukla SK. *Chlamydia pneumoniae* infection in a breeding colony of African clawed frogs (*Xenopus tropicalis*). Emerg Infect Dis. 2000;6:196–9. 10.3201/eid0602.000216 10756157 PMC2640851

[22] Daszak P, Cunningham AA. Anthropogenic change, biodiversity loss and a new agenda for emerging diseases. J Parasitol. 2003. In press.

