# Correction, Vol. 9, No. 8

**DOI:** 10.3201/eid0909.C10909

**Published:** 2003-09

In “Emerging Pathogen of Wild Amphibians in Frogs (*Rana catesbeiana*) Farmed for International Trade,” by Rolando Mazzoni et al., errors occurred in the figure legend on page 996.

The correct caption to the Figure appears below:

Figure. a and b, histopathologic findings from infected frogs. Characteristic sporangia (s) containing zoospores (z) are visible in the epidermis (asterisk, superficial epidermis; arrow, septum within an empty sporangium; bars, 10 μm). c, Skin smear from infected frog, stained with 1:1 cotton blue and 10% aqueous potassium hydroxide (aq KOH) (D, developing stages of *Batrachochytrium dendrobatidis*; arrow, septum within a sporangium; bar, 10 μm). d, Electron micrograph of an empty sporangium showing diagnostic septum (arrow) (bar, 2 μm).

The corrected article appears online at http://www.cdc.gov/ncidod/EID/vol9no8/03-0030.htm

We regret any confusion these errors may have caused.

